# Eight-Port millimeter wave MIMO antenna loaded with novel frequency selective surface for higher data rate transmission

**DOI:** 10.1371/journal.pone.0341823

**Published:** 2026-02-02

**Authors:** Malini Soman, Manish Sharma, Seema Narwal, Tanweer Ali

**Affiliations:** 1 Department of Electronics and Communication Engineering, School of Engineering and Technology, SGT University, Gurugram, Haryana, India; 2 Department of Electrical, Electronics and Communication Engineering, Galgotias University, Greater Noida, Uttar Pradesh, India; 3 Manipal Institute of Technology, Manipal Academy of Higher Education, Manipal, India; Galgotias College of Engineering and Technology, Greater Noida, INDIA

## Abstract

In this research, an eight-port multiple-input-multiple-output (MIMO) antenna integrated with frequency frequency-selective surface (FSS) is presented for 28.0 GHz millimeter-wave (n257/n261) bands. The radiating patch consists of a circular-ring shape with a plus-shaped stub embedded in it. The antenna element is fed by a 50 Ω microstrip feed line and a quarter-wave transformer as a matching network. The MIMO antenna measures 50 mm × 50 mm and achieves spatial diversity through an orthogonal arrangement of eight radiating elements. A 10 × 10 array of FSS is also designed at 28.0 GHz and measures 60 mm × 60 mm. The FSS structure is intended for a band-stop filter and consists of a circular ring with a plus-shaped stub. The proposed MIMO antenna has a peak realized gain of 13.89 dBi and features directional 2-D radiation patterns. The specific absorption rate analysis is also performed, resulting in a value of 0.0274 W/kg at 28.0 GHz.

## 1. Introduction

Recent advancements in planar antennas have introduced several new technologies, including multiple-input-multiple-output (MIMO) schemes for improved diversity, which can be spatial or polarization. MIMO technology allows spatial multiplexing by using numerous antennas at both the transmitter and receiver, increasing capacity without demanding more bandwidth. MIMO also improves signal quality by diversity gain, minimizing the effect of fading and interference, resulting in more reliable links [1,2]. A high-gain antenna is one of the requirements for higher data rate transmission to increase the signal strength and range [3]. Metasurfaces can increase antenna gain by functioning as reflectors or superstrates, controlling radiation effectively and eliminating undesired losses. Metasurfaces offer significant advantages in conventional antenna systems by allowing precise control over electromagnetic waves [4,5]. Metasurfaces can also improve impedance matching, surface wave losses, and mutual coupling in MIMO systems, resulting in improved efficiency and performance. Several forms of metasurfaces are employed in antenna systems to improve radiation performance, including artificial magnetic conductors (AMCs), electromagnetic band gap (EBG) structures, and frequency selective surfaces (FSSs) [6,7]. FSSs can enhance antenna gain by acting as a reflecting or transmissive surface that selectively filters electromagnetic waves. FSS can be used as a ground reflector instead of conventional ground planes, minimizing surface wave losses and improving radiation efficiency. FSS surface can also reduce undesirable side lobes, improving overall antenna performance by directing energy into the desired beam direction [8−9].

A high-gain MIMO antenna with FSS could improve the signal-to-noise ratio (SNR) and minimize path loss and interference. Several studies have been published in the literature that demonstrate the challenges in developing MIMO systems and the integration of FSS. A single antenna system with low gain reduces radiation efficiency due to fading. This issue can be solved by placing numerous identical-radiating elements on the same plane of the substrate, with orientations such as adjacent/orthogonal/mirrored sequences. In [10], a four-port reconfigurable MIMO antenna was proposed with dual-band for IoT and 5G applications, and the configuration included an I-shaped stub to improve isolation. A coplanar waveguide (CPW)-fed rectangular patch antenna with an inverted U-slot and an L-connected stub was designed for 5G bands using liquid crystal polymer in [11]. In [12], a tri-band four-port MIMO antenna was developed using multiple separated L-strips connected to the feedline, with parasitic strips providing high isolation. In [13], a metamaterial structure was used, which was created by printing the antenna on both sides of the surface and using a split ring resonator to provide high isolation. In [14], a modified chamfered rectangular patch with two rectangular stubs and an etched slot was designed for 5G and Wi-Fi bands. Several four-port MIMO antennas with radiators placed orthogonally and partial ground surfaces to achieve operational wide bandwidth, mitigating interference, and achieving high self-isolation were presented [15–18].

Another method of reducing mutual coupling was to use a neutralization line (NZL), which cancels the current vector flowing in opposite directions [19]. The two-element inset-fed rectangular-patch antenna array showed a resonance frequency of 28.0 GHz [20], and the array configuration achieves a maximum peak gain of 11.0 dBi. In [21], the reported MIMO antenna consisted of four MIMO elements structured with eight patches and a cross-shaped defected ground surface (DGS) to provide a high gain. In [22], a six-port MIMO antenna was comprised of slotted ring ground and inset feeding, and I-shaped stubs were used to improve isolation. In [23], an eight-port MIMO antenna was proposed for satellite communication featuring a neutralizer block, parasitic elements, and DGS to decrease mutual coupling. In [24], an eight-element MIMO antenna design was suggested with a T-shaped patch, ring-shaped ground plane, and step-shaped feedline. In [25], an eight-port millimeter-wave MIMO antenna using hybrid approaches was described, which included meander line parasitic and DGS for electromagnetic coupling reduction. In [26], a quad-port MIMO antenna for the 5G millimeter-wave band was described, with an isolating metallic sheet inserted between the patch radiators to increase isolation. In [27], the antenna was reported with loaded stubs and slots, and to increase its performance further, an FSS sheet was placed behind the antenna. In [28], a MIMO antenna array for 5G millimeter-wave applications was constructed, with an FSS used to increase gain. However, the above-reported MIMO antennas showed strong mutual coupling, resulting in lower isolation and poor system performance. Also, the antenna gains were low, and the design complexity was high; therefore, optimizing them takes time and advanced computational approaches. To overcome these difficulties, simple antenna design and modelling approaches are required to assure optimal MIMO antenna performance for real-world millimetre-wave applications. A 28.0 GHz circularly polarized MIMO antenna is formed by placing two radiating elements adjacent to one another and the other two by 180◦ orientation [29], which achieves isolation of more than 20.0 dB. Also, the modal-analysis approach is applied to a flower-shaped MIMO antenna [30] with 10-mode analysis, where CM2, CM4, CM5, and CM6 contribute to the operational bandwidth. Meta-material with double negative characteristics is used in designing of wideband antenna generating impedance bandwidth of 20.22 GHz-30.65 GHz [31,32]. Fractal shape geometry is another physical structure used in radiating-patch design, which generates dual-bands with Band1 = 5.40 GHz-6.82 GHz and Band2 = 8.39 GHz-11.42 GHz [33]. MIMO antenna designed for Cognitive applications covers X/Ku/K-bands, which utilizes a fractal-patch with orthogonal-orientation [34–36] exhibits isolation of more than 20.0 dB, and an eight-port MIMO antenna with 2 × 2 configuration utilizes half-mode substrate integrated waveguide, and its array configuration enhances the peak-realized-gain more than 12.20 dBi [37].

This paper presents an eight-port MIMO antenna integrated with an FSS layer that is intended to offer a band-stop response at 28.0 GHz. The measured operating bandwidth of the antenna is 27.45 − 28.38 GHz, and the transmission coefficient at 28.0 GHz is −66.97 dB, implying that very little power is transmitted between ports A and B. The MIMO antenna uses the adjacent orthogonal sequence to achieve high interelement isolation. The proposed radiating patch and single-element FSS employ a circular ring with embedded plus-shaped stubs. The antenna has a maximum gain of 13.87 dBi and improved diversity performance.

An eight-port multiple-input-multiple-output (MIMO) antenna integrated with a frequency-selective surface (FSS) is presented for the 28.0 GHz millimeter-wave (FR2 n257/ n261) bands. The design uses a circular-ring radiating patch with an embedded plus-shaped stub, a 50 Ω microstrip feed, and a quarter-wave transformer for matching, implemented in a compact 50 mm × 50 mm footprint. The integrated FSS is used not only as a frequency-selective filtering layer but also as an electromagnetic coupling suppression structure to preserve MIMO performance in a dense eight-port layout.

Unique contributions of the proposed FSS-integrated MIMO structure (compared to prior works with similar geometries)

(a) FSS integrated as an active isolation/filter layer (not just a reflector):

Unlike prior ring/plus-stub designs that rely purely on element spacing, decoupling stubs, or neutralization lines, the proposed design embeds an FSS tuned to the 28 GHz passband, which simultaneously suppresses inter-element surface waves and unwanted out-of-band coupling, improving isolation without increasing array footprint.

(b) Dense eight-port topology with preserved MIMO metrics:

Many similar-geometry studies report two- or four-port arrays or require large inter-element gaps. The present work demonstrates an eight-port layout in a compact 50 × 50 mm area while maintaining low mutual coupling, good envelope correlation coefficient (ECC), and diversity gain — enabled by the FSS and careful feed/matching design.

(c) Combined spectral selectivity + radiation/gain improvement:

The FSS is optimized to be frequency-selective at n257/n261 while acting as a partial superstrate that enhances broadside gain and reduces back-radiation, giving a two-fold benefit (filtering + radiation performance) that most prior similar geometries do not deliver concurrently.

(d) Element design that simplifies matching at mmWave:

The circular-ring patch with an embedded plus-shaped stub, coupled with a quarter-wave transformer, provides robust impedance matching across the 28 GHz targeted band. Prior ring/slot or stub designs often require complex matching networks or a larger area to get similar matching at FR2 frequencies; this approach keeps the feed network simple and compact.

(e) Robustness to mutual coupling for adjacent triads of elements:

In the final configuration, three elements are placed adjacent on each side (creating potential high coupling regions). The paper shows how the FSS and element geometry mitigate coupling in these dense groupings, reducing the need for bulky decoupling structures used in earlier work.

(f) Practical fabrication and integration advantages:

The FSS is realized with a manufacturable topology compatible with standard PCB processes and can be colocated above the MIMO plane — easing practical implementation versus more exotic decoupling schemes that require multi-layer, high-precision assembly.

(g) Targeted support for FR2 28 GHz (n257/n261) ecosystem:

The design explicitly targets n257/n261 bands used in contemporary mmWave deployments, optimizing element/FSS resonances for those channels — prior similar geometries are often demonstrated at lower mmWave frequencies or without explicit cellular band targeting.

## 2. Antenna design (Single-Port Analysis)

**Fig 1:**
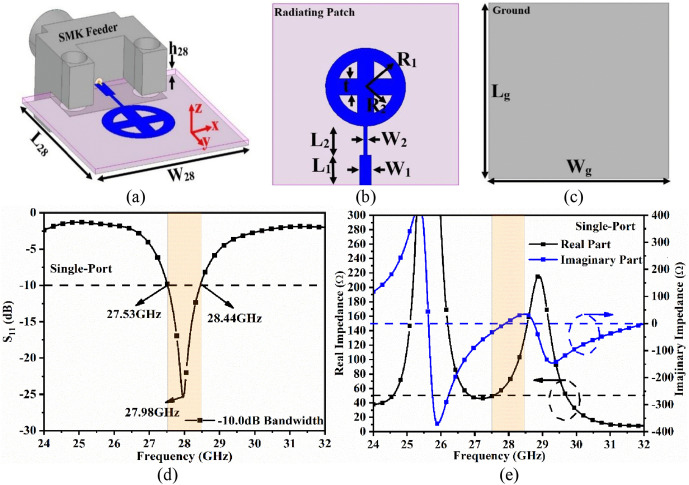
Single-port configuration (a) Oblique view, (b) Front view, (c) Back view, (d) S-parameters, (e) Impedance.

Fig 1 depicts the proposed single-port antenna operating in the n257/n261 millimeter-wave bands. The patch is printed on the top surface of the Rogers RT Duroid 5880 (*ε*_*r*_ = 2.2, tan *δ* = 0.0009) dielectric substrate, and the antenna has a size of *L*_28_ mm × *W*_28_ mm × *h*_28_ mm. The oblique view, front view, and ground view of the antenna structure are shown in Fig 1(a), Fig 1(b), and Fig 1(c), respectively. The microstrip feedline and quarter-wavelength transformer are used to feed power to the patch, and the proposed antenna is fed through an SMK connector.

The patch is a circular ring with an outer radius of *R*_1_ mm and an inner radius of *R*_2_ mm. In addition, a plus-shaped stub with a length of *R*_2_ mm and a thickness of *t* mm is inserted into the circular ring. The microstrip feedline (*L*_1_ mm × *W*_1_ mm) is connected to a quarter-wavelength transformer (*L*_2_ mm × *W*_2_ mm) attached to the circular ring. The ground with dimensions *L*_*g*_ mm × *W*_*g*_ mm is printed on the opposite side of the dielectric substrate. Fig 1(d) depicts the simulated S-parameters of the single-port antenna, with the antenna achieving a bandwidth of 27.53 − 28.44 GHz. Fig 1(e) shows the impedance graph (*R* ± *jX*) Ω, where *R* is plotted on the left and ±*jX* is plotted on the right. The net impedance is (47.23 − *j*0.856) Ω at the 28.0 GHz resonance frequency. Table 1 lists the dimensions of the single-port antenna.

**Table 1:** Dimensions of the single-port antenna.

Parameter	in mm	Parameter	in mm	Parameter	in mm
*L*_28_	20.0	*R*_2_	3.00	*L*_2_	3.20
*W*_28_	20.0	*t*	1.75	*W*_2_	0.40
*h*_28_	0.787	*L*_1_	3.30	*L*_*g*_ = *g*	20.0
*R*_1_	4.25	*W*_*1*_	1.25	*W*_*g*_	20.0

### 2.1. Antenna Evolution

**Fig 2:**
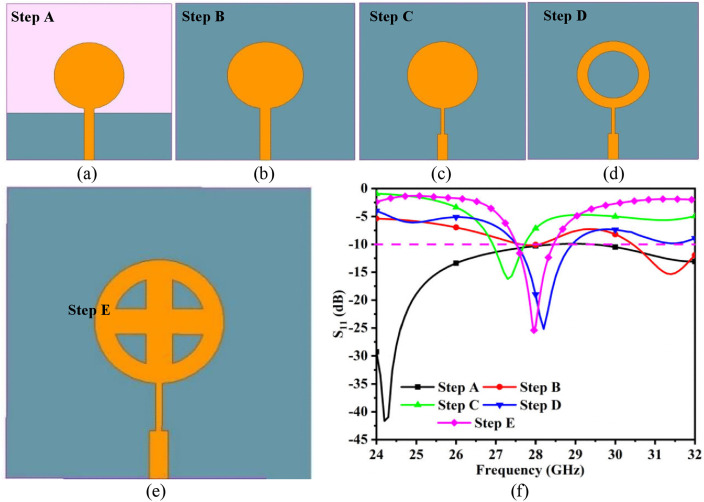
Evolution of single antenna configuration (a) Step A, (b) Step B, (c) Step C, (d) Step D, (e) Step E, (f) S11 for steps A to E.


Fig 2 illustrates the evolution of the single-port antenna. The circular patch with radius *R*_1_ is connected to the microstrip feedline, with partial ground on the opposite surface of the dielectric substrate, as shown in Fig 2(a) (step A), and the radius *R*_1_ of the circular patch can be calculated as [7,13].

$$R_{\text{1}}=\frac{n}{{\left\{\text{1}\;+\;\frac{\text{2}h_{\text{2}\text{8}}}{\pi \varepsilon_{r}n\left[ln\left(\frac{\pi n}{\text{2}h_{\text{2}\text{8}}}\right)\;+\;\text{1}.\text{7}\text{7}\text{2}\text{6}\right]}\right\}}^{\frac{\text{1}}{\text{2}}}}$$ (1)

$$n=\frac{\text{8}.\text{7}\text{9}\text{1}\;\times \;{\text{1}\text{0}}^{\text{9}}}{f_{l}\sqrt{\varepsilon_{r}}}$$ (2)

Fig 2(f) depicts the S-parameters for each design stage. The antenna corresponding to step A has a maximum resonance at 24.20 GHz with S_11_ = −41.63 dB. However, the step A antenna fails to achieve resonance at 28.0 GHz. Therefore, the step A antenna must be modified to achieve antenna B with a complete ground plane as shown in Fig 2(b). The step B antenna uses a 50 Ω microstrip feedline, but there is an impedance mismatch at the circular patch junction. Thus, better matching is required between the feedline and the circular patch, which is accomplished by introducing a quarter-wavelength transformer. Step C in Fig 2(c) demonstrates the ability to address the trade-off between matching impedance between the feedline and the circular patch. Fig 2(d) depicts the step D antenna, which involves the etching of a circular patch, forming a ring structure that achieves −10 dB impedance bandwidth of 27.55 − 28.96 GHz with resonance at 28.20 GHz. Fig 2(e) shows the final antenna design (step E), which includes a plus-shaped stub placed within the circular ring. The proposed single-port antenna offers an operating bandwidth of 27.51 to 28.45 GHz.

### 2.2. Parametric investigation

**Fig 3:**
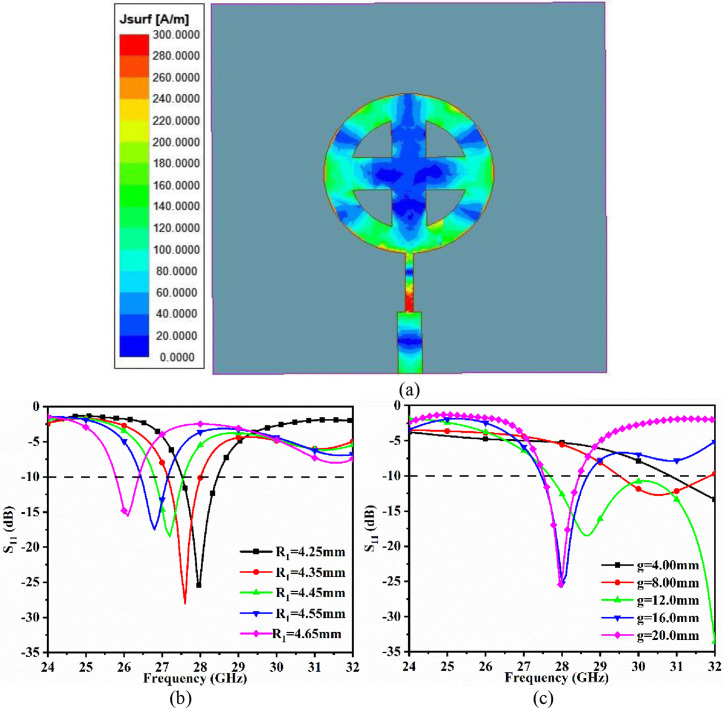
Surface current distribution at (a) 28.0 GHz; Parametric study for (b) R1, (c) g.

Fig 3 depicts the surface current distribution at 28.0 GHz, as well as a parametric study of the key parameters. The distribution indicates that the quarter-wavelength transformer matches the impedance between 50 Ω microstrip and the circular patch, resulting in higher surface current density and maximum signal flow from the feedline to the patch, as shown in Fig 3(a).

The antenna dimensions shown in Fig 1 are obtained by optimizing the key physical parameters such as *R*_1_ (radius of the circular patch) and *g* (ground length). The radius *R*_1_ of the circular patch is increased from 4.25 to 4.65 mm with a 0.1 mm step, as shown in Fig 3(b). The resonance frequency shifts from 27.96 GHz (S_11_ = −25.42 dB) at 4.25 mm to 26.10 GHz (S_11_ = −15.57 dB) at 4.65 mm, as the radius of the circular patch increases, indicating an increase in area. This also indicates that increasing the radius *R*_1_ not only shifts the resonance frequency but also degrades impedance matching.

The length of the ground *g* (*L*_*g*_) is critical not only for achieving the desired bandwidth, but also for improving impedance matching. For *g* = 4 mm, the antenna exhibits an impedance mismatch, resulting in no useful bandwidth. For values of *g* = 8 mm, 12 mm, and 16 mm, impedance matching improves, with *g* = 16 mm achieving a nearly required bandwidth of 27.48 − 28.70 GHz, resonance frequency centered at 28.15 GHz, and S_11_ = −25.32 dB. For *g* = 20 mm, which covers the 400 mm^²^ area on the dielectric surface, the bandwidth is 27.51 − 28.446 GHz while also achieving the required resonance frequency at 27.96 GHz with S_11_ = −24.42 dB.

### 2.3. Equivalent Circuit

The procedure for extracting R, L, and C components is as follows.

(1) The impedance graph (Real and Imaginary) is plotted, which is already shown in Fig 1(e). The total impedance value corresponding to 28.0 GHz is noted, which is (R-jX) Ω=(47.23-j0.856) Ω. This value is also reflected in Table 2.
(2) Using Equation (7), where the imaginary value of Z_11_ (0.856) is known, f_o_ = 28.0 GHz is a known quantity, value of L is calculated.
(3) The value of L obtained from Equation (7) is substituted in Equation (8) to get the value of C.

**Table 2:** RLC values of the antenna at 28.0 GHz.

Frequency (GHz)	*R* ± *jX*		*L* (pH)	*C* (pF)
	Re.	Img.		
28.0	47.23	−0.856	4.89	6.6138

After extracting all three values of R, L, and C, the conceptual equivalent circuit with all the components connected in parallel is connected, excited, and the graph is simulated in ADS.

**Fig 4:**
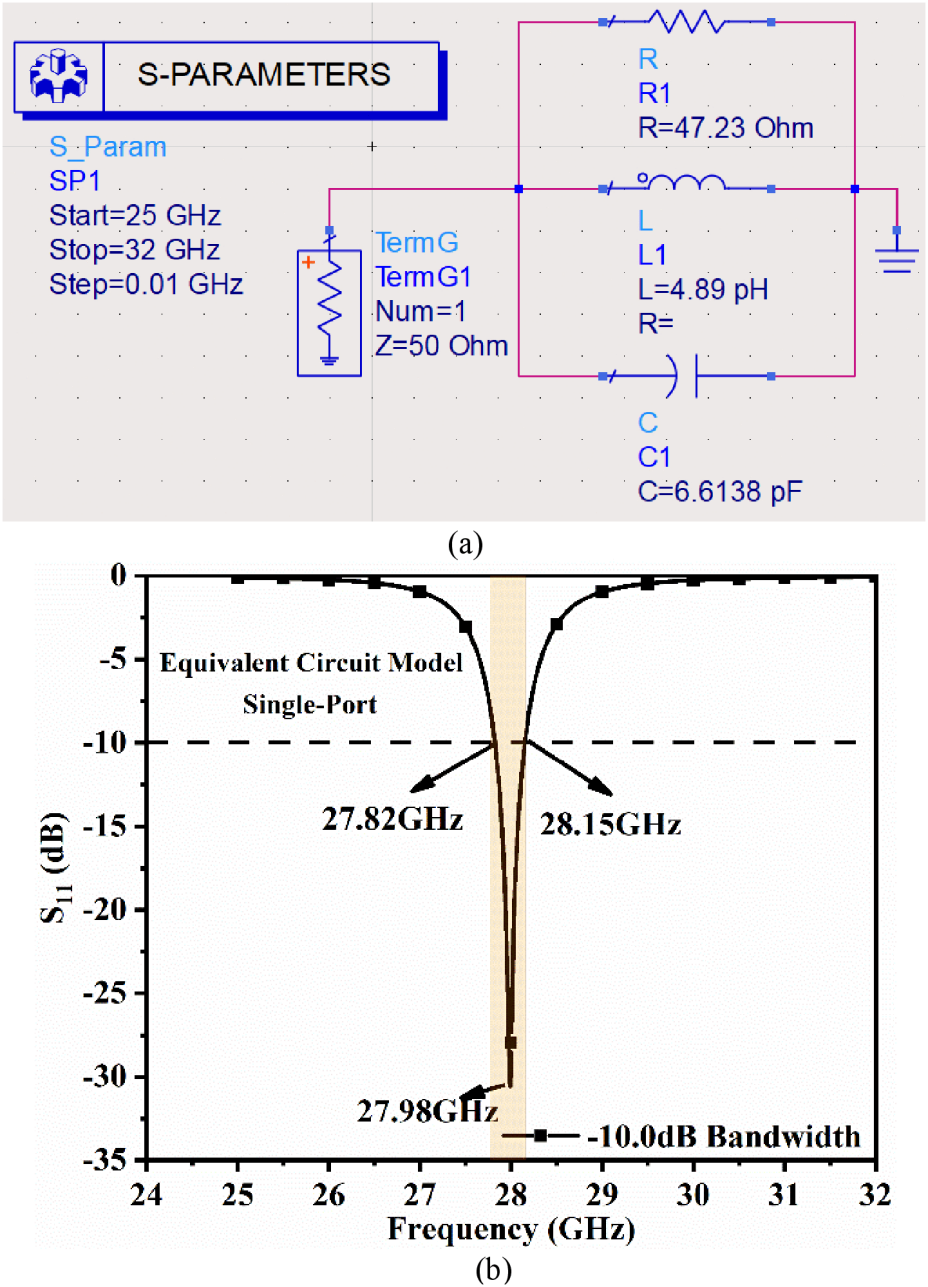
(a) Equivalent circuit model of the antenna element, (b) S11 plot.


The proposed single-port antenna (shown in Fig 1) can also be implemented using circuit theory, and its equivalent circuit model is implemented in Keysight ADS software, as shown in Fig 4. Fig 4(a) shows that the antenna can be considered as a parallel *RLC* circuit that produces resonance at 28.0 GHz. The *RLC* model is terminated by a matched 50 Ω impedance and is excited by an input signal sweep from 25.0 to 32.0 GHz with a step size of 0.01. Equations (3) −(5) are used to calculate the values of inductance (*L*, in pH) and capacitance (*C*, in pF) at the 27.96 GHz resonance frequency, which are listed in Table 2 [38,12].

$$f=\frac{\text{1}}{\text{2}\pi \sqrt{LC}}$$ (3)

$$L=\frac{Img(Z_{\text{1}\text{1}})}{\text{2}\pi f_{\text{0}}}$$ (4)

$$C=\frac{\text{1}}{{\left(\text{2}\pi f_{\text{0}}\right)}^{\text{2}}L}$$ (5)

Fig 4(b) depicts the simulated S-parameters of the antenna, along with detailed values. The antenna achieves resonance at 27.98 GHz, with S_11_ = −30.56 dB and an operating bandwidth of 27.82 − 28.15 GHz. The circuit model theory validates the performance of the proposed single-port antenna, which is suitable for n261 millimeter-wave applications.

## 3. Frequency selective surface (FSS)

### 3.1. Unit cell of the FSS

**Fig 5:**
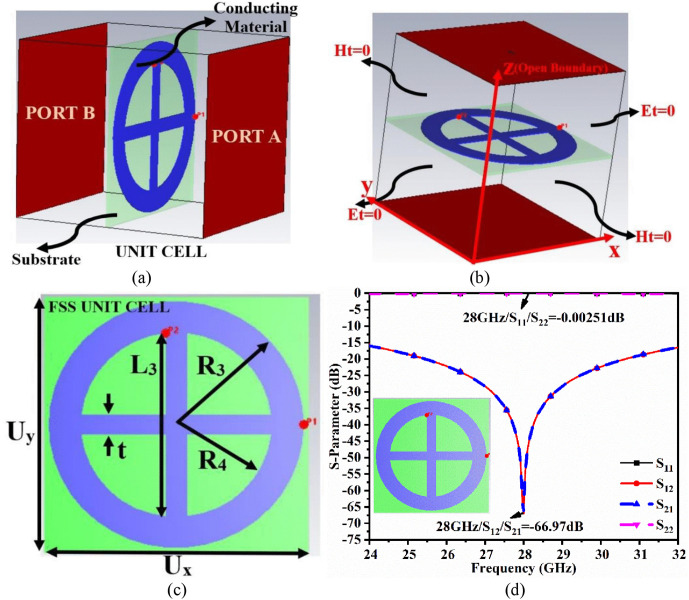
Unit cell of the FSS (a) Port excitation, (b) Boundary conditions, (c) Dimensions, (d) S-parameters.

FSS is a two-dimensional periodic structure with metallic arrays on the dielectric substrate. The design of the FSS structure determines whether the impinged incoming plane wave is transmitted or reflected, partially or completely. FSS, also known as spatial filters, can block or pass electromagnetic waves. Fig 5 depicts the unit cell of the proposed FSS. The FSS is designed on the Arlon AD300C substrate with a permittivity of 2.97, loss tangent of 0.0021, and thickness of 0.10 mm. The substrate size is *U*_*x*_ × *U*_*y*_ = 6 mm × 6 mm with the FSS cells printed on the top surface. The cell is made of a ring structure with an outer radius of *R*_3_ = 2.85 mm and an inner radius of *R*_4_ = 2.30 mm. A plus-sign-shaped stub is embedded within the ring, with an arm measuring 4.60 mm and a thickness of 0.40 mm.

The designed FSS structure is placed within the box, and input port A excites the top surface of the FSS with the patch, while input port B is assigned to the opposite surface of the box, as shown in Fig 5(a). The boundary conditions are applied to the box, with one pair of perpendicular walls assigned as *E*_*t*_ = 0 and the other pair assigned as *H*_*t*_ = 0, as shown in Fig 5(b). Fig 5(c) shows the dimensions of the FSS, while Fig 5(d) depicts the S-parameter analysis of input port A and port B excitation.

The objective of the designed FSS structure is to reflect all signals or act as a band stop filter when all signals are incident on it for the bandwidth specified. Fig 5(d) shows S-parameter analysis with the value of S_11_/S_22_ = −0.00251 dB at 28.0 GHz, indicating that the proposed FSS acts as a band-stop filter and reflects incident signals. S_11_/S_22_ ≅ 0 dB indicates maximum impedance mismatch, which causes maximum reflection of the input signal. On the other hand, Fig 5(d) shows that S_12_/S_21_ = −66.97 dB, indicating that very little power is transmitted from input port A to input port B.

### 3.2. FSS layer

**Fig 6:**
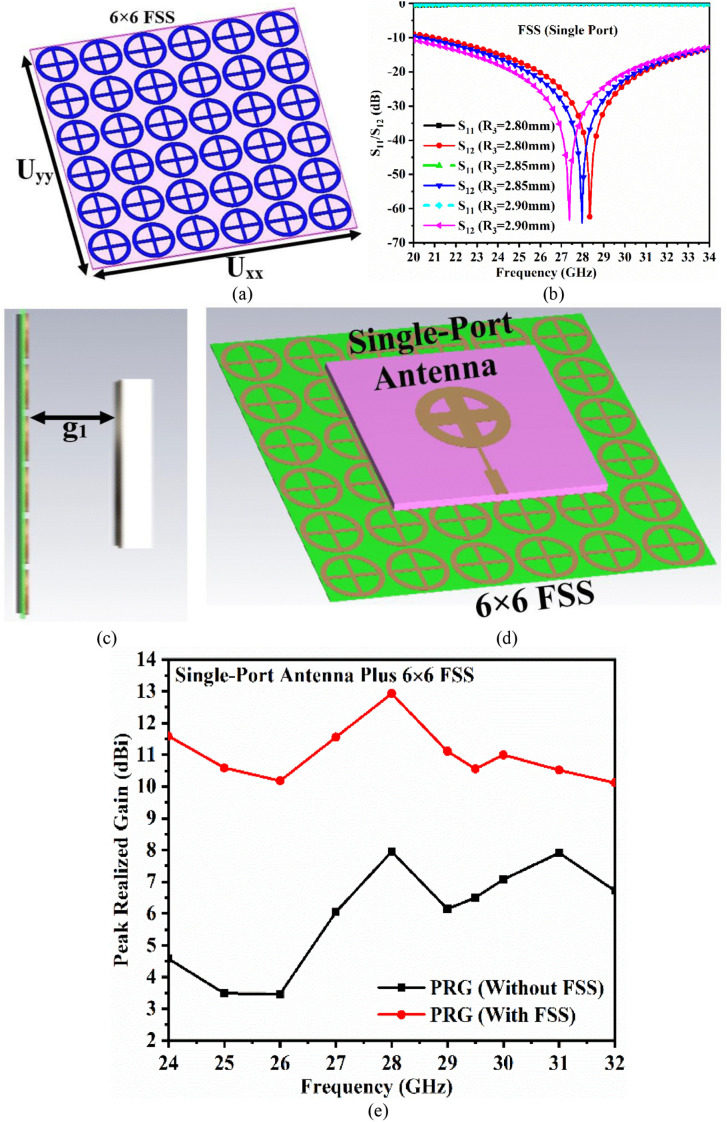
FSS array (6 × 6) (a) Oblique view, (b) Parametric study, (c) Gap between single-port antenna and FSS array, (d) Oblique view of antenna and FSS array, (e) Gain comparison.

Fig 6 depicts the conversion of a single-element FSS to an array. Fig 6(a) depicts the 6 × 6 FSS array with dimensions of *U*_*xx*_ × *U*_*yy*_ = 36 mm × 36 mm. Fig 6(b) depicts the plots of S_11_ and S_12_ for *R*_3_ = 2.80 mm, 2.85 mm, and 2.90 mm. The reflection coefficients for *R*_3_ = 2.80 mm are S_11_ = −0.00264 dB, with S_12_ = −62.38 dB centered at 28.34 GHz. The higher *R*_3_ value of 2.90 mm shifts the frequency to 27.38 GHz, with S_11_ = −0.00296 dB and S_12_ = −63.38 dB. The optimal *R*_3_ value is 2.85 mm at 27.98 GHz, which results in S_11_ = −0.00256 dB and S_12_ = −64.21 dB. This optimizes the physical dimensions of the FSS, making it suitable for use as a reflective surface and integration with an antenna.

In Fig 6(c), a 6 × 6 FSS array designed for 28.0 GHz is placed below the proposed single-port antenna, with a gap of *g*_1_ mm. The gap *g*_1_ is maintained at a distance of *λ*/2 ≤ *g*_1_ ≤ *λ*/4, where *λ* is calculated at 28.0 GHz. The purpose of placing the FSS below the antenna is to reflect the back radiation of the antenna in the opposite direction, allowing for constructive interference with the main lobe, increasing the electric-field intensity (V/m) in the boresight direction, and increasing the antenna gain. Fig 6(e) shows that *g*_1_ = 2.65 mm is the optimal dimension for increasing the gain from 7.95 (without FSS layer) to 12.93 dBi (with FSS layer) at 28.0 GHz.

## 4. MIMO antenna configuration

### 4.1. Two-port MIMO antenna

**Fig 7:**
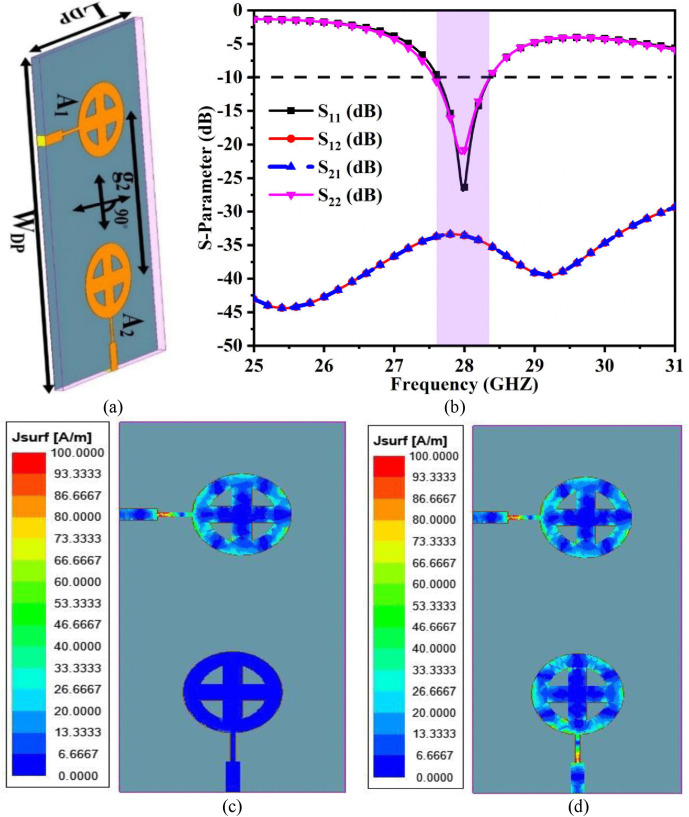
Two-port MIMO antenna (a) Configuration (b) S-parameter results, (c) Surface current density at 28.0 GHz with single-port excitation, (d) Surface current density at 28.0 GHz with dual-port excitation.

When deployed in a wireless communication system, the single-port antenna suffers from destructive interference at the receiver section due to signals arriving from different angles and delays, a phenomenon known as multiple path propagation. This problem can be solved by combining multiple identical radiating patches and forming a MIMO system that achieves diversity schemes such as spatial or polarization. The single-port antenna depicted in Fig 1 is converted into a two-port MIMO antenna shown in Fig 7. Fig 7(a) depicts two identical radiating structures, antenna 1 (A_1_) and antenna 2 (A_2_). The multi-port antenna improves both link reliability and data rate transfer. The spectral efficiency is also increased due to spatial multiplexing utilizing multiple independent data gateways over the same frequency.

Fig 7(a) shows that the two elements A_1_ and A_2_ are placed orthogonally with a spacing of *g*_2_ = 19.20 mm. The overall dimension of the two-element MIMO/diversity antenna is *W*_*DP*_ × *L*_*DP*_ = 20 mm × 40 mm and occupies the full ground. The two-port antenna operates between 27.58 − 28.36 GHz, with the resonance frequency at 27.96 GHz, as shown in Fig 7(b). The isolation between the elements is greater than 33 decibels, indicating that no additional methodology is required to improve isolation.

The surface current density is another way to understand the isolation between neighbouring elements. Fig 7(c) and (d) depict the surface current density of the antenna with single-port (other port terminated by 50 Ω) and two-port excitation. In the first case, the radiating patch, A_1_, efficiently radiates the input signal with no impact or very little mutual coupling between A_1_ and A_2_. In the latter case, A_1_ and A_2_ are fed by the same input signal simultaneously, and it can be seen that they radiate the input signal independently with low mutual coupling.

### 4.2. Four-port MIMO antenna

In order to increase data rate transmission and reduce signal fading, the two-port MIMO antenna is converted to a four-port configuration. The Shannon-Hartley theorem states that increasing the number of radiating elements in the MIMO system increases the bandwidth.

**Fig 8:**
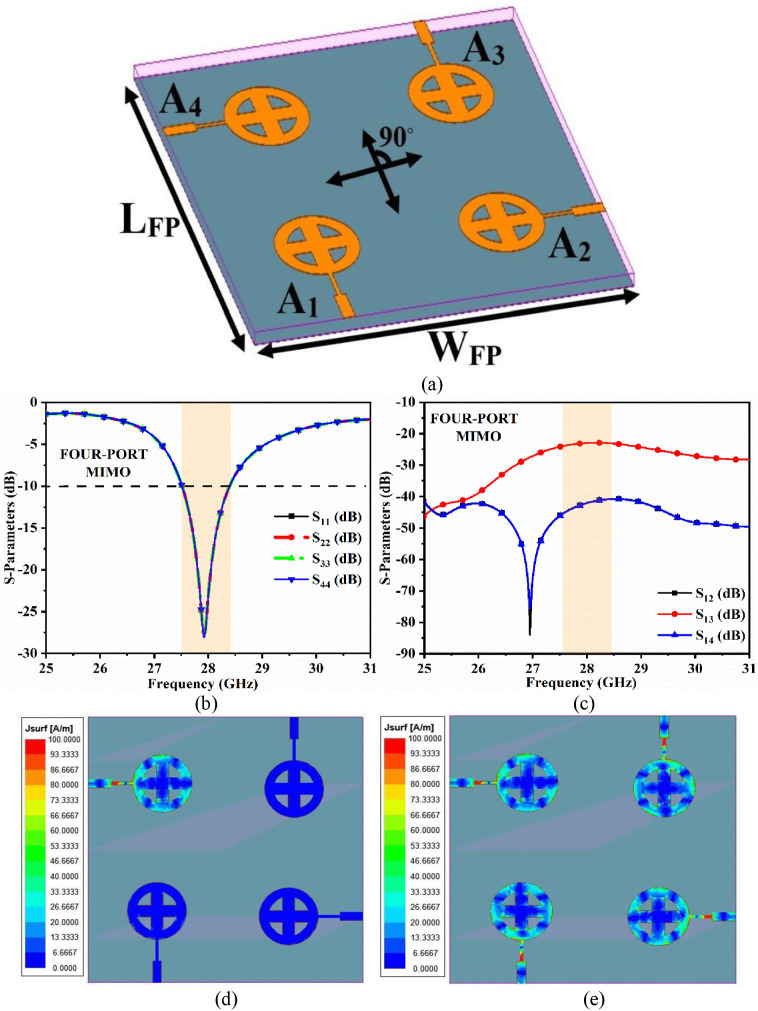
Four-port MIMO antenna (a) Configuration, (b) Reflection coefficients, (c) Transmission coefficients, (d) Surface current density at 28.0 GHz with single-port excitation, (e) Surface current density at 28.0 GHz with four-port excitation.

Fig 8(a) depicts the four-port MIMO antenna system with four identical radiating structures denoted as A_1_, A_2_, A_3_, and A_4_. Here, all four radiating elements are placed in a cyclic and orthogonal sequence, with the dimensions of the MIMO antenna *W*_*FP*_ × *L*_*FP*_ = 40 mm × 40 mm. Fig 8(b) and (c) show the reflection and transmission coefficients of the MIMO antenna, respectively. All radiating structures produce a −10 dB bandwidth of 27.50 − 28.35 GHz, with a resonance frequency centered at 27.99 GHz and S11 = −24.36dB. The isolation between input port 1 (A_1_)−input port 2 (A_2_) and input port 1 (A_1_)−input port 4 (A_4_) is 42.0 dB, while the isolation between input port 1 (A_1_)−input port 3 (A_3_) is 23.0 dB.

Fig 8(d) depicts the surface current distribution for a single-port excitation (A_1_) with ports 2, 3, and 4 (A_2_, A_3_, and A_4_) terminated by 50 Ω, while Fig 8(e) depicts all ports (A_1_, A_2_, A_3_, and A_4_) excited simultaneously. In both cases, isolation is better, and it does not affect the radiation efficiency of individual radiating elements.

### 4.3. Eight-Port MIMO Antenna

The channel capacity theorem, when applied to two-port, four-port, and eight-port MIMO antennas, reduces signal fading while also increasing efficiency by increasing the number of radiating elements.

**Fig 9:**
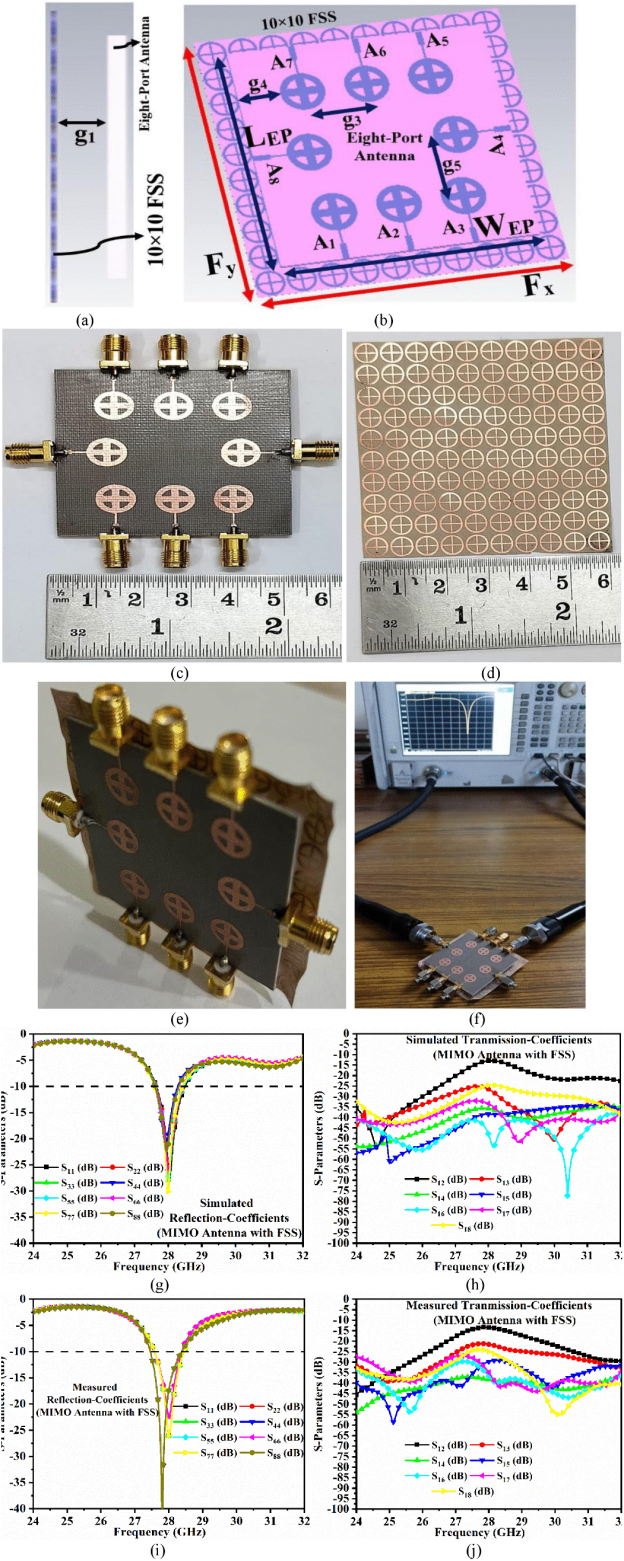
Eight-port MIMO antenna (a) Side view, (b) Slant view with dimensions, (c) Fabricated prototype, (d) Fabricated prototype of 10 × 10 FSS array, (e) Slant view of antenna integrated with FSS, (f) FSS-based MIMO antenna connected to VNA for S-parameters measurement, (g) Simulated reflection coefficients of the FSS-based MIMO antenna, (h) Simulated transmission coefficients of the FSS-based MIMO antenna, (i) Measured reflection coefficients of the FSS-based MIMO antenna, (j) Measured transmission coefficients of the FSS-based MIMO antenna.

Fig 9 depicts the dimensions of the MIMO antenna with integrated FSS as well as a comparison between simulated and measured outcomes. Fig 9(a) depicts the side view of the FSS- FSS-FSS-integrated MIMO antenna with a gap of *g*_1_ = 2.65 mm, which is identical to the gap used in the FSS-integrated single-port antenna. Fig 9(b) shows the schematic of the FSS-integrated MIMO antenna with an overall dimension of *W*_*EP*_ × *L*_*EP*_ = 50 mm × 50 mm. The three identical radiating elements, A_1_, A_2_, and A_3_, are adjacent to each other, with an edge distance of *g*_4_ = 8.20 mm from the substrate and a centre-to-centre spacing of *g*_3_ = 12.50 mm between A_1_ and A_2_. The radiator A_4_ is orthogonal to A_1_, A_2_, A_3_ and A_5_, A_6_, A_7_, with a spacing of *g*_5_ = 9.90 mm between A_3_ and A_4_. Similarly, A_5_, A_6_, and A_7_ are adjacent to each other and opposite to A_1_, A_2_, and A_3_. A_3_ is also orthogonally placed, regardless of the radiating patches A_1_, A_2_, A_3,_ and A_5_, A_6_, A_7_.

Fig 9(a) and (b) show the 10 × 10 FSS layer (*F*_*x*_ × *F*_*y*_ = 60 mm × 60 mm) placed beneath the eight-port MIMO antenna at a distance of *g*_1_. The antenna configuration includes an eight-port MIMO antenna (2500 mm^2^) and a FSS array (3600 mm^2^), both working for the n261 millimeter-wave band. Fig 9(c) and (d) depict the fabricated prototypes of the eight-port MIMO antenna and FSS array. The conventional photolithographic method is used to create prototypes with accurate dimensions. Fig 9(e) shows a 3-D view of the fabricated prototype of the eight-port MIMO antenna integrated with FSS, connected by thin Teflon rods on the edges.

Fig 9(f) shows the validation of the result for the FSS-integrated eight-port MIMO antenna, with the antenna resonating at 27.98 GHz.

The simulated reflection coefficients S_*aa*_ (a = 1, 2, 3, 4, 5, 6, 7, and 8) corresponding to S_11_/S_22_/S_33_/S_44_/S_55_/S_66_/S_77_/S_88_ and transmission coefficients S_*ab*_ (*a* = 1, *b* = 2, 3, 4, 5, 6, 7, and 8) corresponding to S_12_/S_13_/S_14_/S_15_/S_16_/S_17_/S_18_ are plotted in Fig 9(g) and (h). The simulated bandwidth ranges from 27.645 − 28.50 GHz, with the maximum resonance recorded at 27.99 GHz and S_77_ = −30.32 dB. Fig 9(h) shows that the transmission coefficients exceed 12 dB for all the port combinations of S_*ab*_ (*a* = 1, *b* = 2, 3, 4, 5, 6, 7, and 8). The measured S-parameters are plotted in Fig 9(i) and (j), with a measured −10 dB bandwidth corresponding to 27.456 − 28.38 GHz. When compared to simulated reflection coefficients, the curves show nominal deviation, which can be due to fabrication tolerances such as etching and alignment errors, SMK connector loss, and feed misalignments. It can also be noted that the S_12_ parameter nearly grazes the −10.0dB at 28.0 GHz resonance, which indicates the isolation between port1-port2. However, the resonance of 28.0 GHz and the bandwidth generated from Element 1 & Element 2 are not compromised in both simulated result (Fig 9(g)) & measured result (Fig 9(h)).

#### 4.3.1. Diversity Performance of Eight-Port MIMO Antenna.

**Fig 10:**
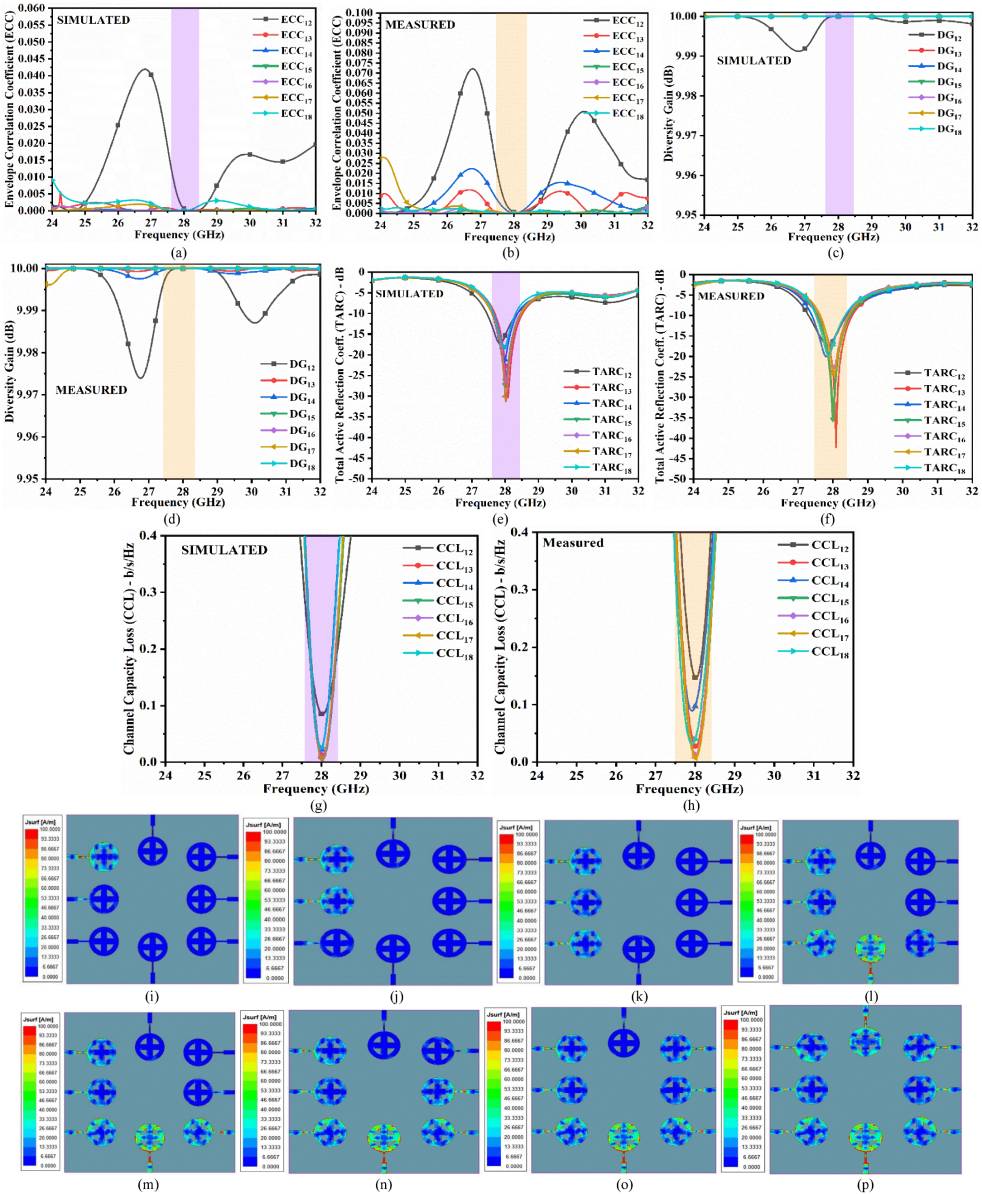
Diversity performance analysis (a) Simulated ECC, (b) Measured ECC, (c) Simulated DG, (d) Measured DG, (e) Simulated TARC, (f) Measured TARC, (g) Simulated CCL, (h) Measured CCL, (i) Surface current density (SCD) with port 1 excitation, (j) SCD with ports 1 and 2 excitation, (k) SCD with ports 1, 2, and 3 excitation, (l) SCD with ports 1, 2, 3, and 4 excitation, (m) SCD with ports 1 to 5 excitation, (n) SCD with ports 1 to 6 excitation, (o) SCD with ports 1 to 7 excitation, (p) SCD with ports 1 to 8 excitation.

Fig 9 depicts the eight-port MIMO configuration, which discusses the arrangement of radiating elements with integrated FSS and the S-parameter results. However, the interaction between each of the radiating elements is further investigated by studying the simulated/measured diversity performance parameters such as envelope correlation coefficient (ECC), diversity gain (DG), total active reflection coefficient (TARC), and channel capacity loss (CCL), as shown in Fig 10.

All parameter calculations for ECC, DG, TARC, and CCL are performed between input port 1 and port 2, input port 1 and port 3, input port 1 and port 4, input port 1 and port 5, input port 1 and port 6, input port 1 and port 7, and input port 1 and port 8. The correlation between adjacent MIMO antennas is investigated by calculating ECC between any two ports as [13,14,48],


$$ECC=\rho_{e}(m,s,N)=\frac{{\left|\sum_{n\;=\;\text{1}}^{N}S_{m,n}^{*}\;S_{n,s}\right|}^{\text{2}}}{\pi_{k\;=\;(m,s)}\left[\text{1}\;-\;\sum_{n=\text{1}}^{N}S_{m,n}^{*}\;S_{n,k}\right]}$$ (6)

Equation (6) shows the calculation of ECC between the *m*th and *s*th port, which is calculated as,


$$ECC=\frac{{\left|S_{\text{11}}^{*}S_{\text{12}}+S_{\text{12}}^{*}S_{\text{22}}{+S}_{\text{13}}^{*}S_{\text{32}}+S_{\text{14}}^{*}S_{\text{42}}\right|}^{\text{2}}}{\left(\text{1}-{\left|S_{\text{11}}\right|}^{\text{2}}-{\left|S_{\text{21}}\right|}^{\text{2}}-{\left|S_{\text{31}}\right|}^{\text{2}}-{\left|S_{\text{41}}\right|}^{\text{2}}\right)\left(\text{1}-{\left|S_{\text{12}}\right|}^{\text{2}}-{\left|S_{\text{22}}\right|}^{\text{2}}-{\left|S_{\text{32}}\right|}^{\text{2}}-{\left|S_{\text{42}}\right|}^{\text{2}}\right)}$$ (7)

The variation of ECC ranges from 0 to 1, indicating no interference. This is the ideal case, with perfect impedance matching and each MIMO radiating element effectively isolated from the others. However, in practice, this condition is difficult to achieve, and standard values of less than 0.50 must be obtained. The simulated and measured ECC are calculated between input port 1 and port 2 (ECC12), input port 1 and port 3 (ECC13), input port 1 and port 4 (ECC14), input port 1 and port 5 (ECC15), input port 1 and port 6 (ECC16), input port 1 and port 7 (ECC17) and input port 1 and port 8 (ECC18). Fig 10(a) and Fig 10(b) show that the simulated ECC values are less than 0.00025, while the measured values are less than 0.005.

The diversity gain (DG) is calculated to quantify the performance characteristics of the chosen diversity scheme, as well as to represent the quality and reliability of the MIMO elements. The DG is linked to the ECC as [13,14,48],


$$DG=\text{1}\text{0}\sqrt{\text{1}-{\left|\rho_{e(n\text{2}\text{6}\text{1})}\right|}^{\text{2}}}$$ (8)

The standardized value for DG is ~ 10 dB. For the proposed eight-port MIMO antenna configuration integrated with FSS, the simulated and measured DG values correspond to 9.99 dB and ~10.0 dB, as noted from Fig 10(c) and (d).

The ratio involved between the total reflected power (TRP), irrespective of the radiating elements, and the total power impinged on the radiator is known as TARC, which is calculated from Equations (9)−(13) [13,14,37] for an eight-port MIMO antenna integrated with FSS.


$${𝛤}_{a}^{t}=\frac{Available\;Power\;(AP)\;-\;Radiated\;Power\;(RP)}{Available\;Power\;(AP)}$$ (9)

$$TARC=\frac{\sqrt{\sum_{i=\text{1}}^{N}{\left|m_{i}\right|}^{\text{2}}}}{\sqrt{\sum_{i=\text{1}}^{N}{\left|s_{i}\right|}^{\text{2}}}}$$ (10)

where [*m*] and [*s*] represent incident and reflected power in dB. TARC for phase values of S-parameters is calculated as,


$$b_{\text{1}}=S_{\text{11}}a_{\text{1}}+S_{\text{12}}a_{\text{2}}=S_{\text{11}}a_{\text{0}}e^{j\theta_{\text{1}}}+S_{\text{12}}a_{\text{0}}e^{j\theta_{\text{2}}}=a_{\text{1}}\left(S_{\text{11}}+S_{\text{12}}e^{j\theta }\right)$$ (11)

$$b_{\text{2}}=S_{\text{21}}a_{\text{1}}+S_{\text{22}}a_{\text{2}}=S_{\text{21}}a_{\text{0}}e^{j\theta_{\text{1}}}+S_{\text{22}}a_{\text{0}}e^{j\theta_{\text{2}}}=a_{\text{1}}\left(S_{\text{21}}+S_{\text{22}}e^{j\theta }\right)$$ (12)

$${TARC}_{n\text{2}\text{6}\text{1}}=\frac{\sqrt{{\left|S_{\text{11}}+S_{\text{12}}e^{j\theta }\right|}^{\text{2}}+{\left|S_{\text{21}}+S_{\text{22}}e^{j\theta }\right|}^{\text{2}}}}{\sqrt{\text{2}}}$$ (13)

The simulated and measured TARC values are calculated for port 1 and port 2, port 1 and port 3, port 1 and port 4, port 1 and port 5, port 1 and port 6, port 1 and port 7, and port 1 and port 8, resulting in less than −10.0 dB for FSS-integrated MIMO antenna as shown in Fig 10(e) and Fig 10(f). Furthermore, as shown in Fig 9(g) and Fig 9(i), the impedance matching is not as good in the simulation scenario as it is in the measurement. Therefore, total outgoing power is reduced regardless of total incident power, gradually lowering the values of TARC.

When the proposed antenna is deployed in a wireless communication environment, the signal transmitted between *T*_*x*_ and *R*_*x*_ suffers some loss in the MIMO system, as evidenced by a loss of bits in the channel of less than 0.40 b/s/Hz, which is assessed by [13,14,48],


$$CCL=-{log}_{\text{2}}det\left(\alpha^{s}\right)$$ (14)

where


$$\rho_{mm}=\text{1}-\sum_{n\;=\;\text{1}}^{\text{4}}{\left|S_{mn}\right|}^{\text{2}}$$ (15)

$$\rho_{ms}=-\left(S_{mm}^{*}S_{ms}+S_{sm}^{*}S_{ms}\right)$$ (16)

Fig 10(g) and Figure 10(h) show the simulated and measured CCL values for the eight-port MIMO antenna integrated with FSS, which are less than 0.20 b/s/Hz and 0.38 b/s/Hz, respectively.

Fig 10(i) to Fig 10(p) show the surface current density for the eight-port MIMO antenna integrated with FSS, which shows the excitation of a particular port and the remaining port terminated by a 50 Ω load. Table 3 presents a summary of the surface current density for various port excitation combinations in cyclic order. Overall, the radiation efficiency remains consistent with the −10.0 dB bandwidth for both simulated and measured S-parameters.

**Table 3:** Surface current density for the eight-port MIMO antenna integrated with FSS.

Port Excitation	Terminated Port	Observation
Input port 1	Input port 2, Input port 3, Input port 4, Input port 5, Input port 6, Input port 7, Input port 8	No interference was observed between neighboring antennas A_2_ and A_8._
Input port 1, Input port 2	Input port 3, Input port 4, Input port 5, Input port 6, Input port 7, Input port 8	No interference was observed between neighboring antenna A_3._
Input port 1, Input port 2, Input port 3	Input port 4, Input port 5, Input port 6, Input port 7, Input port 8	Low interference was observed between neighboring antennas A_4_ and A_8_
Input port 1, Input port 2, Input port 3, Input port 4	Input port 5, Input port 6, Input port 7, Input port 8	No interference was observed between neighboring antennas A_5_ and A_8_
Input port 1, Input port 2, Input port 3, Input port 4, Input port 5	Input port 6, Input port 7, Input port 8	No interference was observed between neighboring antennas A_6_ and A_8_
Input port 1, Input port 2, Input port 3, Input port 4, Input port 5, Input port 6	Input port 7, Input port 8	No interference was observed between neighboring antennas A_7_ and A_8_
Input port 1, Input port 2, Input port 3, Input port 4, Input port 5, Input port 6, Input port 7	Input port 8	No interference was observed between neighboring antenna A_8_
Input port 1, Input port 2, Input port 3, Input port 4, Input port 5, Input port 6, Input port 7, Input port 8	None	No interference was observed between all radiating elements

Table 3 explains the relevance when the port/ports are excited with other ports terminated and their impact on the neighbouring radiating elements as shown in Fig 10(i) to Fig 10(p). The following cases are detailed below

Case 1: Input port 1 is excited, and the remaining ports are terminated (Input port 2-Input port 8), which shows that no interference is observed between the neighbouring radiating elements.

Case 2: The two input ports, Input port 1 & Input port 2, are excited with the remaining ports terminated by 50Ω. Also, it is observed that no interference is recorded with the adjacent antenna A3.

Case 3: The three input ports, Input port 1-Input port 3, are excited, which also has no impact or interference on the antenna elements, A4 & A8.

Case 4: The excitation of Input port 1-Input port 4, with the remaining four ports terminated, also records no interference.

Case 5: With five ports excited (Input port 1-Input port 5), the impact in the form of interference on A6 & A8 is also negligible.

Case 6: Records the excitation of Input port 1-Input port 6 with Input port 7 & Input port 8 terminated by 50Ω. This case records no interference impact on antennas A7 & A8.

Case 7: The seven-port excitation, other than the Input port 8, also records minimal interference on Antenna A8.

Case 8: Finally, all the port excitations shown in Fig 10(p) also show minimal interference, but the isolation is high, which does not impact the performance of individual radiating elements.

#### 4.3.2. Far-Field Performance of Eight-Port MIMO Antenna.

The far-field analysis of the proposed eight-port MIMO antenna with an integrated 10 × 10 array of FSS is crucial for understanding the behavior of radiation characteristics in modern wireless communication systems. The far-field distance (FFD) is defined as,


$$FFD\geq \frac{\text{2}\;\times \;{W_{EP}}^{\text{2}}}{\lambda_{\text{2}\text{8}\;GHz}}$$ (17)

**Fig 11:**
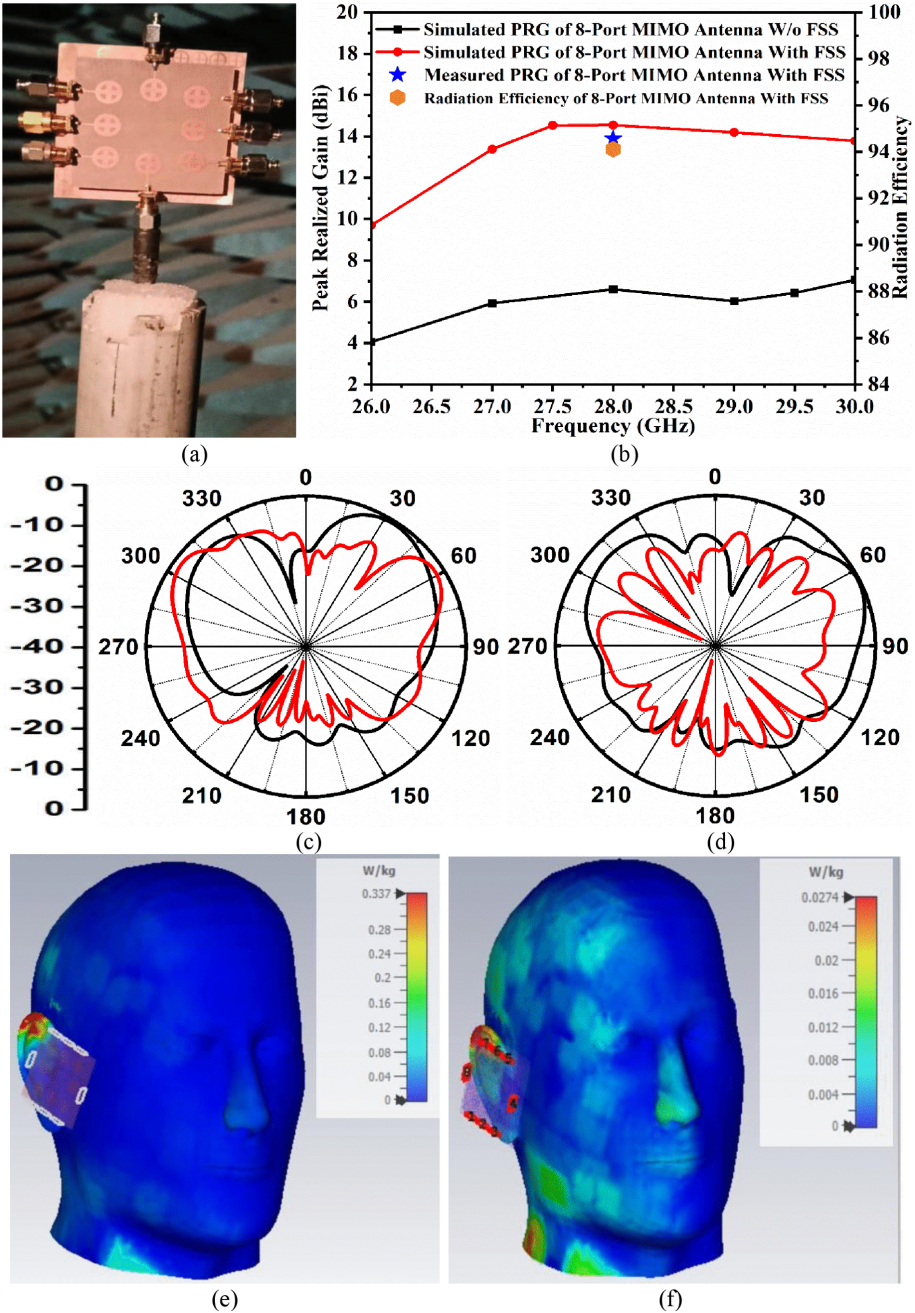
Far-field measurement (a) Antenna prototype inside the anechoic chamber, (b) Peak gain and Radiation efficiency, (c) 2-D radiation pattern at 28.0 GHz in elevation plane, (d) 2-D radiation pattern at 28.0 GHz in azimuth plane, (e) Surface current density for eight-port MIMO antenna without FSS, (f) Surface current density for eight-port MIMO antenna with FSS.

Fig 11(a) depicts the fabricated prototype of the eight-port MIMO antenna integrated with FSS placed within the anechoic chamber for far-field measurements. Equation (15) shows the shortest distance between the transmitter and the receiver that allows the test antenna to be in the far-field. The far-field distance is determined by the maximum dimension of the antenna (*W*_*EP*_ = *L*_*EP*_) and the wavelength that corresponds to 28.0 GHz (n261). Fig 11(b) depicts the peak realized gain values for the eight-port MIMO antenna with and without FSS integration (simulated and measured). The MIMO antenna without FSS has a maximum peak realized gain of 6.60 dBi. The goal of FSS integration is to increase the peak realized gain by reflecting signals and algebraically adding the electric field density to the main lobe. The simulated peak realized gain for the FSS-integrated eight-port MIMO antenna is 14.54 dBi, whereas the measured values are 13.89 dBi at 28.0 GHz. Fig 11(b) also records the radiation efficiency of the proposed work with a value corresponding to 84.12% at 28.0 GHz resonance frequency.

Fig 11(c) and (d) show the simulated and measured 2-D radiation patterns at 28.0 GHz for the elevation plane (E-plane) and azimuth plane (H-plane). The locus shape of the patterns is affected by material properties and conductor losses at 28.0 GHz, which are more directional in both planes. The pattern is directive in the elevation plane, with suppressed back lobes due to the presence of FSS.

However, there is very close agreement between the simulated and the measured results, but the minimal deviations are due to reasons including fabrication tolerances, variation in the material property, effects introduced due to the connector, and the feeding effects with imperfection soldering, imperfect measurement setup limitations, and anechoic chamber imperfections due to residual reflections from absorbers.

#### 4.3.2. Specific absorption rate (SAR) analysis of eight-port MIMO antenna.

When the proposed antenna is used in proximity to the human body, the electromagnetic interference between the antenna and the human head phantom model must be calculated. The three layers, skin, fat, and muscle, are incorporated into the phantom model to investigate the effects of radiation from the FSS-integrated eight-port MIMO antenna placed near the head. The tissue model has a thickness of 2.0 mm for skin, 5.0 mm for fat, and 5.0 mm for muscle. Table 4 shows the electromagnetic properties of the tissue model, which include the required electrical permittivity, conductivity, loss tangent, and density at a frequency of 28.0 GHz. These values are used to calculate the specific absorption rate (SAR) using Equation (18),

**Table 4:** Electrical properties of tissue model at various frequencies [39,40].

Tissue	Frequency (GHz)	Electrical Permittivity	Conductivity	Loss Tangent	Density (Kg/m^3^)
Skin	28.0	16.5	25.8	1.0016	1109
Fat	28.0	6.09	5.06	0.29471	911
Muscle	28.0	24.4	33.6	0.88284	1090


$$SAR=\frac{\sigma |E{|}^{\text{2}}}{\rho }$$ (18)

where *σ* is the conductivity of the body tissue (S/m), *E* is the applied electric field (V/m), and *ρ* is the mass density of the body tissue (kg/m^3^).

The SAR analysis is conducted using two scenarios: one with the eight-port MIMO antenna placed 5.0 mm away from the human phantom model in the absence of FSS (Fig 11(e)) and the other with integrated FSS (Fig 11(f)). Table 5 displays the simulation results for both scenarios. For SAR analysis, a power of 1 W is applied to 1 g of tissue, which should be less than 1.6 W/kg. According to the observations, the maximum SAR value at 28.0 GHz without FSS is 0.337 W/kg, while the maximum value with integrated FSS is 0.0274 W/kg. The FSS placed between the human-head phantom and the eight-port MIMO antenna reduces the value of SAR. The FSS is designed for a band-stop filter centered at 28.0 GHz, preventing back-lobe radiation from reaching the human-head phantom.

**Table 5:** SAR values of the eight-port MIMO antenna without and with FSS.

Frequency (GHz)	SAR (W/Kg) Without FSS	SAR (W/Kg) With FSS
28.0	0.337	0.0274

## 5. Performance comparison

Table 6 compares the proposed FSS-integrated eight-port MIMO antenna with existing antenna designs. Reference [2] calculates the SAR at resonance frequency values, but the gain is extremely low, as no technique was used to increase the gain. The proposed antenna achieves a peak realized gain of 13.89 dBi by integrating a 10 × 10 FSS array beneath the antenna. Also, as an eight-port MIMO antenna configuration, the antenna is smaller than other configurations. In comparison to other published works, the proposed work inherits several characteristics, such as better diversity, higher peak gain, and better SAR value at 28.0 GHz.

**Table 6:** Comparison of the proposed work with existing antenna designs.

Ref.	Size (mm × mm)	No. of Ports	Operating Bandwidth (GHz)	FSS Array	FSS Size (mm × mm)	Max. Peak Gain (dBi)	ECC/ DG (dB)	Isolation (dB)	SAR (W/Kg)
[10]	40 × 40	4	1.0-3.50	NO	NO	5.30	<0.08 9.50	>40.0	NO
[11]	56 × 56	4	2.54-3.56 4.28-4.97 5.37-8.85	NO	NO	3.20	<0.13 9.95	>20.0	1.94 at 3.50 GHz 0.78 at 4.70 GHz 0.46 at 6.50 GHz
[12]	50 × 50	4	3.18-20.10	NO	NO	4.85	<0.20 9.95	>20.0	NO
[16]	50 × 50	4	2.47-3.38 4.94-7.24	NO	NO	5.21	<0.064 9.95	>15.0	NO
[18]	45 × 50	4	23.4-35.0	NO	NO	12.4	14.1 × 10^−4^ 9.80	>60.0	NO
[21]	43.6 × 43.6	4	27.5-28.35 37.37-37.6	NO	NO	13.7	<0.05 9.95	>25.0	NO
[22]	38 × 83	6	3.92-5.20	NO	NO	6.45	<0.002 9.98	>15.0	NO
[23]	60 × 60	8	14.0-18.0	NO	NO	6.32	<0.008 9.96	>25.0	NO
[24]	22 × 17	8	26.0-28.0	NO	NO	4.64	<0.005 9.97	>20.0	0.00349 at 27.0 GHz
[25]	54 × 54	8	23.3-27.6	NO	NO	8.60	4 × 10^−4^ 9.99	>26.0	NO
[26]	20.5 × 20.5	4	25.21-32.34	NO	NO	6.82	<0.005 9.97	>20.0	NO
[27]	40 × 30	1	3.9-9.5	5 × 5	58 × 28	9.50	NA NA	NA	NO
[28]	38 × 36	4	27.25-28.85	7 × 7	45 × 45	8.60	<0.002 9.99	>26.0	NO
[35]	50 × 50	4	28 33 38	NO	NO	9.6 7.8 13.7	15 × 10^−4^ 9.998	>25.0	NO
[36]	42 × 36	4	27.30-28.40	NO	NO	10.11	<0.001 9.999	>25.0	NO
Prop.	50 × 50	8	27.456-28.38	10 × 10	60 × 60	13.89	<0.005 9.99	>15.0	0.0274 at 28.0 GHz

## 6. Conclusion

An eight-port MIMO antenna integrated with the FSS is investigated for 28.0 GHz wireless applications. The radiating structure of the single-port antenna and the FSS unit cell are circular rings with embedded plus-shaped stubs. The eight-port MIMO antenna has a measured −10.0 dB impedance bandwidth of 27.4 − 28.38 GHz, with an FSS band-stop filter centered at 28.0 GHz. Spatial diversity is achieved by arranging radiating elements in orthogonal sequences. The diversity parameters have values of ECC < 0.005, DG > 9.99 dB, TARC <−40.0 dB, and CCL ~ 0.386 b/s/Hz. A peak realized gain of 13.896 dBi is recorded at 28.0 GHz resonance frequency, and SAR values equal to 0.0274 W/Kg.

The orthogonal placement of radiating elements ensures effective spatial diversity, while the FSS enhances isolation and contributes to overall system efficiency. Owing to its compact geometry and stable radiation characteristics, the presented architecture also offers strong potential for future extension toward emerging 6G wireless systems, where reconfigurable, beam-steerable, or intelligent surface-based antenna solutions will be essential for enabling higher data rates, ultra-low latency, and adaptive coverage. Additionally, the modular arrangement of the radiating elements and the FSS layer offers a promising foundation for developing beam-steerable architectures, where phased-array techniques or tunable FSS units can enable dynamic beam shaping and enhanced spatial multiplexing for future 6G communication environments.
